# Genetic Characteristics of Brazilian Patients with MH History

**DOI:** 10.3390/genes16101127

**Published:** 2025-09-25

**Authors:** Helga C. A. Silva, Daniela C. Mendonça, Brandow W. Souza, Joilson M. Santos, Lucas S. Souza, Antonio F. R. Junior, Felipe T. G. R. Vasconcelos, Pamela V. Andrade, Acary S. B. Oliveira, Mariz Vainzof

**Affiliations:** 1 Brazilian Malignant Hyperthermia Unit, Department of Anesthesiology, Pain and Intensive Care, Federal University of São Paulo, São Paulo 04039-032, SP, Brazil; 2 Human Genome and Stem Cells Research Center, Institute of Biosciences, University of São Paulo, São Paulo 05508-090, SP, Brazilmvainzof@usp.br (M.V.); 3 Department of Neurology, Federal University of São Paulo, São Paulo 040039-032, SP, Brazil

**Keywords:** malignant hyperthermia, ryanodine receptor calcium release channel, myopathy

## Abstract

Background/Objectives: Malignant hyperthermia (MH) is a pharmacogenetic hypermetabolic syndrome triggered by halogenated agents/succinylcholine. Most families present variants in the *RYR1* and, rarely, in other genes (*CACNA1S*/*STAC3*/*ASPH*). However, each country or region presents differences in the type and frequency of MH variants. Objective: To present the genetic characteristics of Brazilian individuals with MH history. Methods: We reviewed clinical and laboratory data from all families referred for evaluation in the Brazilian MH unit due to a personal or family history of MH during anesthesia. Demographic and clinical data were collected, as well as serum creatine kinase (CK) levels, in vitro contracture test (IVCT) results, and the results of anatomopathological studies of skeletal muscle. Molecular analysis was performed using whole-exome sequencing (WES). Patients with and without variants were compared. Results: WES analysis was available for 61 patients (29 patients who survived an MH crisis and 32 relatives). Variants in the *RYR1* were found in 38 patients (62.2%), and no variants were identified in 20 patients (32.7%). More than one variant in the *RYR1* was found in six individuals. Variants in the *CACNA1S* were found in three patients (4.9%), all of them with concomitant variants in the *RYR1*. Three patients presented variants in the *STAC3* (4.9%). Comparing the groups of patients with variants in the *RYR1* with the one with no variants in this gene, it was observed that the first group showed higher levels of serum CK, a greater frequency of ptosis, strabismus, and cores, and a higher amplitude of contracture in the IVCT after caffeine or halothane. Conclusion: In this preliminary evaluation of Brazilian individuals with MH history, the frequency of *RYR1* variants was similar to those of previous reports in other countries, but there was a higher frequency of *STAC3* and *CACNA1S* variants.

## 1. Introduction

Malignant hyperthermia (MH) is a pharmacogenetic hypermetabolic syndrome expressed as an acute crisis after exposure of susceptible individuals (MHS) to halogenated agents and/or succinylcholine. The prevalence of MH crisis is variable, from 1:10,000 in children to 1:50,000 in adults [1]. MH susceptibility has been associated with genes linked to calcium metabolism and coding proteins of the excitation–contraction coupling complex of the skeletal muscle, with a frequency of 1:217–1:2750 in the general population [1]. Most families present variants in the *RYR1* (50–60%). Rare families present variants in the *CACNA1S* (1%), *STAC3* (<1%), or *ASPH* [1,2].

Due to genetic heterogeneity and the possibility of polygenic inheritance, the gold standard in MH susceptibility diagnosis is the phenotypical in vitro contracture test (IVCT) for the European or the caffeine-halothane contracture test (CHCT) for the North American groups [3]. Currently, only a negative contracture test could exclude MH susceptibility, while a negative genetic test could not. However, efforts have been made to improve the detection capacity of the molecular test, which could be a less invasive diagnostic tool. On the other hand, variants in the *RYR1* are very frequent in the population, and the identification and classification of these variants as pathogenic need additional tests and curation. Only 72 pathogenic or probably pathogenic variants in the *RYR1* are recognized according to the European Malignant Hyperthermia Group (EMHG) list (https://www.emhg.org/genetic-scoring-matrix; accessed on 18 August 2025), and, in addition to the EMHG criteria [3], there is the ClinGen MHS variant curation expert panel (VCEP) criteria [4].

Each country or region presents differences in the type and frequency of variants linked to MH [5,6,7,8,9,10,11,12,13,14,15,16]. The frequency of variants reported in patients with MH ranges from 37% to 87.5% [8,13], with the *RYR1* variants p.R614 and p.G2434R being the most frequent [5,7]. However, there are no genetic population data from South America, except for the description of isolated cases and families. Brazil is the biggest country in South America, and is also characterized by a very mixed population [17]. Beyond the native original people, Brazil was colonized by a European country, Portugal, and received, over time, groups from Africa, Asia, the Middle East, and other European countries such as Germany, Italy, and Spain. Therefore, our main objective was to analyze and characterize the genetic pattern of MH in Brazil.

## 2. Materials and Methods

The Institutional Research Ethics Committee approved this research, and all participants provided their written informed consent. We reviewed the data from all families referred for evaluation in the Brazilian MH unit, in order to select those with a personal or family history of MH linked to anesthesia.

**Figure 1:**
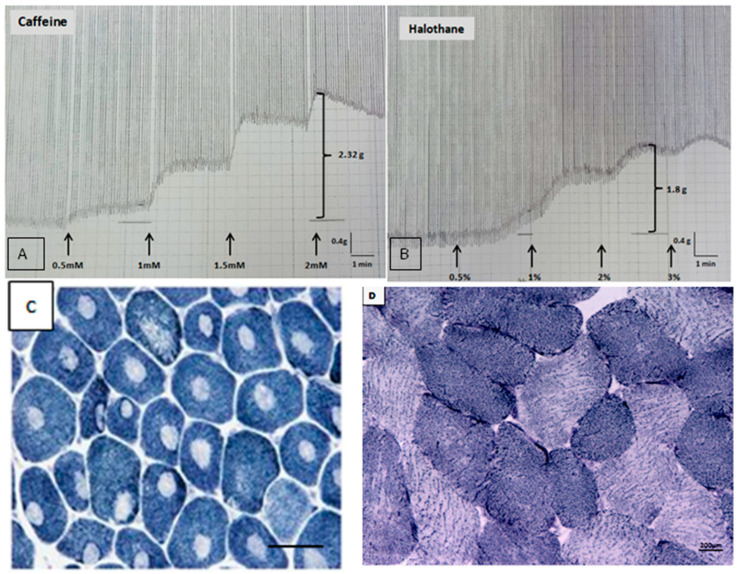
In vitro contracture test and muscle histochemistry. Legend: (**A**,**B**) positive in vitro contracture test for caffeine (**A**) and halothane (**B**) in a patient with a variant of unknown significance (VUS) in exon 51 of the *RYR1* (p.Gly2733Cys; c.8197G > T). (**C**) Cross-sectional section of frozen skeletal muscle showing type I predominance and areas devoid of oxidative activity (cores) inside all type I muscle fibers (NADH, 400×, scale bar: 500 μm, Nikon Eclipse 50i/Nikon DS-Fi1 microscope, Tokyo, Japan) in a patient with a *RYR1* VUS in exon 6 (c.C452A; p.P151Q). (**D**) Cross-sectional section of frozen skeletal muscle showing normal distribution of type I and II muscle fibers and absence of areas devoid of oxidative activity (cores) (NADH, 400×, scale bar: 200 μm, Nikon Eclipse 50i/Nikon DS-Fi1 microscope, Tokyo, Japan) in a patient not susceptible to malignant hyperthermia (negative in vitro contracture test).

Demographic and clinical data were collected, and results of serum creatine kinase (CK), in vitro contracture test (IVCT) according to the EMHG protocol [1], and anatomopathological study of skeletal muscle biopsy with histochemistry were analyzed (Figure 1) when available. Positive IVCT results were reported as MHShc when contractures developed both to halothane and caffeine, MHSh when contractures developed only to halothane, and MHSc when contractures developed only to caffeine. Characteristics of patients with and without variants were compared.

## 3. Molecular Analysis

The DNA of the patients was extracted from peripheral blood lymphocytes using an automated magnetic bead-based method on the QIAsymphony platform, with the QIAsymphony DNA Midi Kit (catalog no. 931255, Qiagen, Hilden, Germany). The genetic investigation was carried out by next-generation sequencing, using first a customized panel including *RYR1* and an additional 95 genes associated with neuromuscular diseases (NMD). After, to expand our investigation, we began to use the Illumina TruSight One Expanded panel, which targets more than 6700 genes and exonic regions that were associated with a described clinical phenotype. Complete exome sequencing was carried out using next-generation sequencing (NGS), using the library preparation and capture kit SureSelectQXT V6 Reagent Kit (Agilent, Santa Clara, CA, USA), and the sequencing reaction was carried out in a HiSeq2500 sequencer (Illumina, San Diego, CA, USA). The generated fastq files were aligned against the GRCh38/hg18 version of the human genome using the BWA-MEM tool (bwakit 0.7.15). Picard Tools 2.18.7 was used in the conversion of SAM files to BAM files and to mark PCR duplicates. The genotypes were called following the GATK best practices, and we used the Genome Analysis Toolkit (GATK) UnifiedGenotyper, version 4.0.9.0. The ANNOVAR tool (https://annovar.openbioinformatics.org/en/latest/, accessed on 18 August 2025) was used for variant annotation. The search for pathogenic mutations was done by analyzing both annotated VCF and BAM files.

Variants were filtered and compared to control populations of 1000 Genomes, NIH, gnomAD, 6500 Exome Sequencing Project (Washington University), and the recently created Online Archive of Brazilian Mutations—AbraOM (http://www.abraom.ib.usp.br/, accessed on 18 August 2025). Rare variants were checked in the *RYR1* (OMIM#180901) and analyzed using bioinformatic tools. Pathogenic variants that were already described were checked in the gene mutations databases HGMD, LOVD, and Clinvar. The pathogenicity of de novo variants was analyzed in prediction sites, including Mutation Taster, Predict SNP1, CADD, DANN, FATHMM, FunSeq2, GWAVA, VEP, SIFT, Polyphen2, and Human Splicing Finder 3.0.

For each variant, we presented the classification according to the specific MH classification from the EMHG [3] (https://www.emhg.org/genetic-scoring-matrix, accessed on 18 August 2025), the VCEP [4], the ACMG [18], and the Association for Clinical Genomic Science (ACGS) [https://www.acgs.uk.com, accessed on 18 August 2025] criteria. All criteria classify the variants in five groups: benign, probably benign, variant of unknown significance, probably pathogenic, and pathogenic. In ACMG classification, each group is linked to criteria for benign (strong and supporting) and pathogenic (very strong, strong, moderate, supporting) variants. The ACMG criteria consider data from population databases, computational and predictive evidence of impact on the gene product, functional studies, segregation data, de novo descriptions, and allelic information. The ACGS criteria combine ACMG criteria with other sources, such as ClinGen, to complement its variant classification.

The EMHG and VCEP criteria support pathogenicity using functional studies with evidence of gain-of-function, which is expressed by increased intracellular calcium release in vitro or ex vivo and a clinical reaction that is consistent with MH under anesthesia and confirmed by a positive IVCT. In EMHG classification, each group of variant classification is linked to specific criteria for benign (stand alone, strong, and supporting) and pathogenic (strong, moderate, supporting) variants. However, the gain-of-function studies are not available so far for many recently described variants.

When variants were not present in the EMHG or VCEP lists, we searched previous descriptions in LOVD.

Our data were analyzed for normality using the K–S distance test. Categorical data were described as absolute (n: number) and relative (%) frequency, and continuous and semi-continuous data were described as mean ± standard deviation or median (25–75% percentiles). The chi-square test was used to compare categorical data, and the unpaired *t*-test or Mann–Whitney test were used to compare continuous and semi-continuous data. A *p*-value of 0.05 was considered for all studies.

## 4. Results

### Clinical and Laboratory Findings in 61 Patients

**Figure 2:**
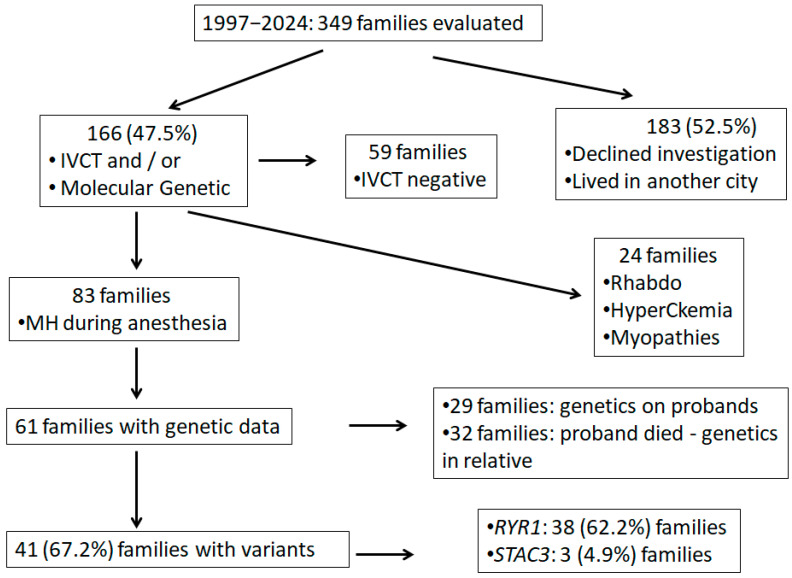
Flowchart of this study. Legend: IVCT: in vitro contracture test, MH: malignant hyperthermia.

From 1997 to 2024, 349 families were referred to evaluation in our MH unit, and 166 of them were already investigated for MH susceptibility with the IVCT and/or molecular analysis (the other declined investigation or lived in another city distant from the investigation center) (Figure 2). Among the 166 investigated families, 59 presented negative results according to the IVCT. Among the other 107 families, 24 were investigated due to rhabdomyolysis, idiopathic hyperCKemia, or myopathies, and were not included in this study. The other 83 had a history of MH crisis linked to anesthesia and were included in this study. A WES molecular analysis was available for 61 of these 83 families, and was performed in 29 patients who survived an MH crisis and 32 relatives, when the proband had died.

This group of 61 patients that underwent NGS analysis had a predominance of men (n = 36) over women (25), with a mean age of 30.8 ± 16.4 years (Supplemental Table S1). The ethnic background was predominantly Caucasian (67.2%). The median basal CK level was 185.5 (87–380) IU/l (n = 54). On neurological examination, ptosis and/or strabismus were present in 24 (39.3%) patients, muscle weakness in 8 (13.1%), and muscle hypertrophy in 31 (50.8%). Cores were found on 8 (15.6%) of the 51 skeletal muscle histochemistry analyses. IVCT was performed in 50 patients (26 MHShc, 17 MHSh, 7 MHSc). The median muscle contracture after exposure to halothane was 0.6 (0.2–2.3) g and that after exposure to caffeine was 0.4 (0–1.9) (n = 50). The 11 patients not submitted to the IVCT had variants in *RYR1* (patients 4, 29, 31, 45, 46, 60) and/or *CACNA1S* (patient 30), *STAC3* (patients 2, 3, 26), *CLCN1* (patient 4), and *DMD* (patient 5).

No variants were found in 20 of the 61 patients with personal or family antecedents of MH crisis (32.7%), while variants in genes related to MH were found in 41 (67.2%) patients: variants in the *RYR1* were found in 38 patients (62.2%) and variants in the *STAC3* were found in 3 patients (4.9%). Three patients with *RYR1* variants also presented *CACNA1S* variants (4.9%). No patient presented variants in the *ASPH*. Overall, variants pathogenic, probably pathogenic, or VUS were present in 37 of 61 patients (60.6%). 

Variants already described in MH were found in 17 patients (Table 1). The variants were previously classified by EMHG or VCEP criteria as pathogenic (n = 7), probably pathogenic (n = 2), VUS (n = 4), and benign (n = 2). In this article, benign variants were included for information purposes only. Analysis of these variants using the data from this report would allow the promotion of two variants that were previously reported. Patient 4 also had a *CLCN1* variant (exon3:c.C316G:p.L106V), probably benign, and presented an MH crisis with an MH Clinical Grading Scale (CGS) score [19] of 63 (rank: 6, almost certain MH crisis).

Multiple variants in the *RYR1* were found in six individuals, five of which had a myopathic phenotype (Supplemental Table S1), including one (patient 8) with a homozygous VUS that was already described (Table 2). Three myopathic patients presented variants that were not previously described, alone (patient 60) or which were associated with one VUS (patient 10) or one benign described variant (patient 5). Patient 5 also had a *DMD* deletion (exons 5–44) and presented an MH crisis with an MH CGS score of 33. Patients 1 (clinically asymptomatic) and 12 (myopathic) presented novel variants associated with variants that were previously described in MH, respectively: one VUS for patient 1, and one that was pathogenic and three that were probably benign for patient 12. The three probably benign variants of patient 12 (p.Ile1571Valp, Arg3366His, and p.Tyr3933Cys) were previously described in a triplet of *RYR* variants associated with MH when alone, and with MH and core-rod myopathy when associated with the variant p.Val4849Ile, which was also present in this patient [20].

Among the 15 variants found in association in these six patients, 8 were previously classified by the EMHG or VCEP criteria as pathogenic (n = 1), VUS (n = 3), probably benign (n = 3), and benign (n = 1). The novel variants would be classified, if they were isolated, as VUS (n = 4), probably benign (n = 1), and benign (n = 2).

Variants that were not previously described in MH were found alone in 12 patients (Table 3). We classified these variants in the *RYR1* based on the criteria established by the EMHG and VCEP that are specific for *RYR1*. The 11 variants were classified as probably pathogenic (n = 1), VUS (n = 8), probably benign (n = 1), and benign (n = 1).

Variants in the *RYR1* were found with concomitant variants in the *CACNA1S* in three families, where the evaluation was performed in one clinically asymptomatic relative in one family, and in the proband in two families (Table 4). The two probands presented ptosis and muscle hypertrophy. Only one of the variants in the *RYR1* was previously reported as pathogenic for MH, and none of the *CACNA1S* variants were previously reported in MH. If they were not associated, the two *RYR1* novel variants would be classified, if they were isolated, as VUS, and the same is true for the three *CACNA1S* novel variants.

The three probands of the families with variants in the *STAC3* presented MH crises and myopathic features such as motor development delay, muscle weakness, contractures, elongated facies, eyelid ptosis, scoliosis, and cryptorchidism. The details of the MH crises of these probands were previously described [21]. The *STAC3* variant was the same in all three unrelated non-consanguineous Afro Brazilian families (c.851G > C: p.Trp284Ser), being present in a homozygous state in the probands (Table 4).

**Figure 3:**
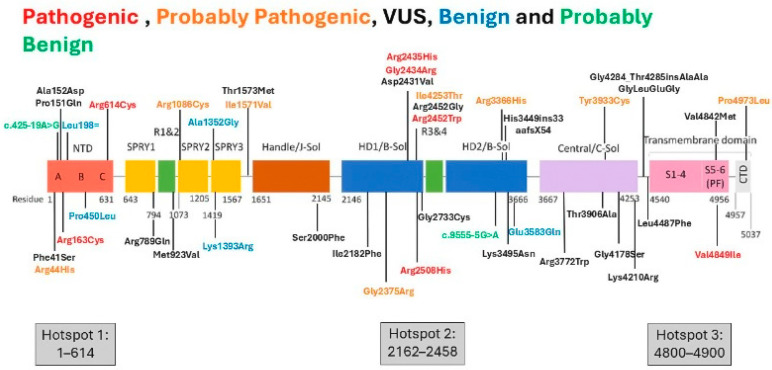
Distribution of variants along the *RYR1* (pathogenic, probably pathogenic, variants of unknown significance, probably benign, benign) found in the Brazilian sample (adapted from Foo et al., 2022 [22]).

The 40 variants in the *RYR1* were distributed along the entire gene, not restricted to hotspots (Figure 3) [22]. Patients with diagnostic variants for MH (14 with pathogenic (11 *RYR1*/3 *STAC3*), 2 with probably pathogenic (*RYR1*)) represented 39% of the 41 patients for which *RYR1/STAC3* variants were found. There were a few recurrent variants in the *RYR1*: the p.R614C and p.V4849I pathogenic variants were each present in three unrelated patients and the p.G2733C VUS was present in two unrelated patients.

Comparing the groups with and without variants (Table 5), we observed that there were no differences regarding age, sex, ethnic background, personal vs. family MH crisis antecedent, or muscle weakness or hypertrophy. The group with variants had higher levels of serum CK, a greater frequency of ptosis and/or strabismus, greater frequency of cores in their muscle histochemistry analysis, and a higher amplitude of contracture in the IVCT after caffeine or halothane. There was a greater frequency of patients with variants in the IVCT positive group after both caffeine and halothane, while there was a greater frequency of patients without variants in the IVCT positive group after only halothane.

## 5. Discussion

This first WES analysis of a cohort of Brazilian patients with personal or family history of an MH crisis linked to anesthesia disclosed variants pathogenic, probably pathogenic, or VUS in 37 individuals (60.6%), predominantly in the *RYR1*. An advantage of this study is the use of exome sequencing. Our study would be better compared with similar recent studies where next-generation sequencing approaches were used to analyze the coding sequences of *RYR1* and *CACNA1S*, such as those from England, North America, and Taiwan [6,7,13]. Other studies presented in this discussion used previous technologies to analyze the entire *RYR1* [5,8,10,11,14,15,16], the 16 *RYR1* hot spot exons [5], and/or the known mutations in *CACNA1S* [5].

Other groups that used a similar methodology (IVCT) presented variable frequencies. Gilles et al., 2015, analyzed the *RYR1* and *CACNA1S* in 62 MH Australian patients (positive IVCT) and found 23 (37%) with known causative or novel variants [8]. Klinger et al., 2014, in a multicenter European study of 200 patients with MH history confirmed by IVCT, described 103 (51.5%) patients with *RYR1* and one with *CACNA1S* (0.5%) variants [5]. Heytens et al., 2019, reported 34 Belgian MH families (positive IVCT), with *RYR1* variants being found in 25 (73%) of them [10]. Miller et al., 2018, in a study of 722 MH British families (confirmation by IVCT or pathogenic variants), found potentially pathogenic variants in 555 of them (76.8%), with no pathogenic variants in *RYR1*, *CACNA1S*, or *STAC3* in 103/722 of the MH families (14%) [7]. Galli et al., 2006, in 50 Italian MH subjects, found 31 variants in 43 patients (86%), with stricter inclusion criteria (positive IVCT for both halothane and caffeine) [15].

Other groups with different methodologies also present variability in the frequency of variants found in MH. Sambuughin et al., 2005, in a study of 30 North American patients (28 CHCT positive, 2 with personal anesthetic MH crisis history), found variants in 21 of them (70%) [16]. Sadhasivam et al., 2019, in another North American study of 38 patients with MH history and positive CHCT, found *RYR1* or *CACNA1S* variants in 30 of them (79%) [6]. Kraeva et al., 2011, found *RYR1* or *CACNA1S* variants in 31 of 36 (86%) Canadian MH families, but used more strict selection criteria, including only patients with a CGS score > 35, or relatives with positive CHCT or abnormal levels of serum CK [11]. Ibarra et al., 2006, described *RYR1* variants in 26 of 46 (56.5%) Japanese patients with MH (Ca-induced Ca release (CICR) on skinned muscle fibers), when excluding 12 patients without personal or family MH crisis antecedents [14]. Yeh et al., 2021, found causative variants in seven of eight (87.5%) MH families (CGS rank ≥ 5) from Taiwan [13].

This variability in the frequency of variants reported in multiple MH cohorts, despite being affected by selection criteria and methodologies, also reflects the genetic background of each country. Our Brazilian sample was characterized by a high genetic variability, which was expressed by the presence of few recurrent variants among the 40 *RYR1* variants reported here, with two of them found in a maximum of three families (p.R614C and p.V4849I). The p.R614C variant was also one of the more frequent in Canada and Europe, which testifies to the European presence in Brazil, but the p.V4849I variant was not reported so frequently in other series [5,11]. In Europe, the most frequent *RYR1* variant was also the R614C, which was found in 25 of 103 families [5]. However, Broman et al., 2015, in 28 Scandinavian index patients with MH, found the *RYR1* p.Arg2435His variant to be the most frequent (n = 8) [12]. Among the 18 variants found in 25 Belgian MH families, the most frequent was the *RYR1* p.Gly341Arg (n = 7) [10]. In England, the *RYR1* variant Gly2434Arg was reported in 118 families (16%) [7]. In Italy, the most frequent *RYR1* variants were the p.Thr2206Met, p.Gly2434Arg, p.Arg3903Gln, and p.Arg4737Trp (n = 3 for each) variants [15]. In Canada, the most frequent *RYR1* variants were p.Arg614Cys (n = 4), p.Gly341Arg (n = 3), and p.Gly2434Arg (n = 4) [11]. In Japan, the most frequent *RYR1* variants were the p.R2508C and the p.R2508H (n = 2 each) variants [14]. In Taiwan, the *RYR1* variant p.Y522C (n = 3) was the most frequent [13].

In 17 of our patients, the identified variants were previously described, mainly with the criteria of EMGH or VCEP. Considering that we are identifying additional families with the same variants and also phenotypically characterized with the positive IVCT tests, it was possible to reclassify two of them. Two variants reached the criteria of possibly pathogenic (patient 54, exon2: c.131G > A (p.Arg44His) and patient 6, exon45: c.7292A > T (p.Asp2431Val)). In the other four families, the classification remained the same, but with additional data increasing their original score (Table 1, patients 9, 11, 48, and 50).

Among the newly described variants, six met the functional PS4 criteria, which increases the likelihood of pathogenicity, especially for that located in exon 46 (p.Arg2452Gly), which is a four-point VUS, just two points short of being reclassified as likely pathogenic. Another notable case is the variant in exon 51 (p.Gly2733Cys), a four-point VUS that both meets the PS4 criteria and has a high REVEL score (PP3_mod), which further supports its potential pathogenicity. The remaining VUS included those with low REVEL scores (BP4_sup), and one variant to which no criteria could be applied, despite a high REVEL score of 0.81. One variant, located in exon 44 (p.Gly2375Arg), was classified as probably pathogenic (PP). The combination of the functional PS4 criteria with PP3_mod and PM1_sup supported this classification. In contrast, two variants were classified as benign (B) and probably benign (PB). These variants met criteria such as BS1_str, BS2_str, and BP4_mod, which supports their non-pathogenic nature and demonstrates the robustness of the classification framework for both risk and reassurance.

Another impressive characteristic of MH samples is the high frequency of novel and private variants reported in only one family. This was the case for the 20 novel variants among the 40 (50%) *RYR1* variants reported here, of which 18 were private and only 1 was present in two families (8197G > T; p.G2733C). The variant c.7354C > G; p.R2452G occurred in the same position as another EMHG pathogenic variant but with a substitution by another nucleotide and amino acid (c.7354C > T; p.R2452W). Around the world, the frequency of novel variants was highly variable: 26/158 (16.4%) in England, 14/51 (27.5%) in European families, 8/18 (44%) in Belgium, 9/18 (50%) in North America, 16/31 (51.6%) in Italy, 15/27 (55.5%) in Canada, 18/29 (62%) in Australia, and 26/33 (78.8%) in Japan [5,7,8,10,11,14,15,16].

The *RYR1* variants c.122T > C; p.F41S and c.2366G > A; p.R789Q, despite not having been previously described in association with anesthetic MH crisis, were reported in conditions related to MH susceptibility. The first one was reported in one compound heterozygous myopathic patient whose parents were not investigated [23]. The second was reported in a patient with hyperCKemia [24].

The presence of more than one variant has been described in some patients, which has been proposed to modify their clinical expression [8] with, theoretically, both protective and deleterious effects. In addition to the functional characterization of the isolated variants, another future field of study would be the definition of the effect of interaction among multiple variants. The frequency of patients with more than one variant in the *RYR1* and *CACNA1S* in our Brazilian sample (8/41: 19.5%) is an important warning against the isolated use of the genetic test, as more than one variant can occur in some families. Compound heterozygous variants were present in 4/103 (3.9%) in European patients, 32/555 (5.8%) in British families, 2/25 (8%) of Belgian families, 5/31 (16%) of Canadian families, 5/26 (19%) of Japanese patients, and 5/23 (22%) of Australian families [5,7,8,10,11,14]. Interestingly, the Australian group described one patient with five *RYR1* variants, who had cores but was clinically asymptomatic [8]. We also identified one patient with five variants in the *RYR1*. Three of their variants (in exons 33, 67, and 85) were combined with the pathogenic variant p.Val4849Ile. Clinically, our patient presented with muscle weakness, ptosis, and cores on muscle biopsy. Another patient with this same genotype, with the pathogenic variant in compound heterozygosity, was susceptible to MH with a positive IVCT, and presented with congenital myopathy with cores and rods on muscle biopsy [20].

Another patient from our sample (patient 1) presented two variants segregating together (exon101:c.14524G > A (p.Val4842Met) and intron68:c.10348-6C > G; p.His3449ins33aafsX54). This same genotype has already been described in other populations, such as in 11 South African patients with a severe form of autosomal recessive myopathy and ophthalmoplegia [25] and in two sibs from Chile with severe neonatal hypotonia [26]. Our patient, in contrast, presented a milder phenotype, with muscle hypertrophy, normal serum CK levels, normal muscle biopsy, and a positive IVCT. According to the authors, this splice site variant results in the production of an aberrant transcript that includes intron 68 and introduces a premature stop codon, but the penetrance of this mutation is incomplete, which results in the expression of both spliced and unspliced transcripts [26]. Wilmshurst et al. (2010) hypothesized that this allele determines the phenotype by two interrelated mechanisms: by reducing the amount of the RyR1 protein and through the V4842M substitution on residual protein [25]. Haplotype analysis indicated a founder effect in the South African population, despite the description from Chile [25,26].

Distribution of variants across all parts of the *RYR1* was present in our sample and has been reported by other groups [7,8], which demonstrates that the variants are not restricted to the described three hot spot regions that exert more influence on the ryanodine receptor type 1 activity, and considered as clusters of variants in the past. Even so, a concentration of variants in these shot spots was reported by the Italian group, with 77.42% of variants being located inside the three hot spots.

The Brazilian group of patients with variants was different from the group without variants. Although the frequency of cores on muscle biopsy in our Brazilian patients was low regarding the 48% observed in Japanese patients (Ibarra), our group with variants presented a higher frequency of cores, myopathic features such as ptosis and/or strabismus, and higher levels of serum CK. Additionally, the group with variants presented a higher amplitude of contracture in the IVCT after caffeine or halothane. Interestingly, variants expressed as MH with cores [27] are associated with marked sensitivity to caffeine, while some associated only with MH have contractures just above the threshold or with different responses between halothane and caffeine [28]. Similar to our findings, Galli et al., 2002, reported a higher frequency of variants in individuals with MHShc (29.8%) versus those with MHSh (11.8%) [9]. Figueroa et al. [29] proposed that patients with MHSh results would be different from those with MHShc results, as variants in *RYR1* and *CACNA1S* were also present in a minority of their MHSh patients, which indicates that this phenotype may be due to mutations in other genes linked to the excitation–contraction process that have not yet been described.

The limitations of this study are related to the difficulty of establishing the ethnic background of a very diverse population, such as the Brazilian population, where the color of the skin does not reflect the genetic background [30]. Then, the indication of Caucasian or Afro Brazilian ethnic background should be approached with reserve. Similarly, the higher frequency of variants in the *CACNA1S* and *STAC3* could be a result of the higher percentage of Afro-descendants [7,21]. Additionally, as pointed out by Miller et al., 2018, comparison among different countries also has limitations that are linked to the in vitro (IVCT, CHCT, and CICR) and genetic methods used for establishing MH diagnosis [7].

## 6. Conclusions

In this preliminary Brazilian sample, the frequency of *RYR1* variants was similar to the results of previous reports in other countries. However, regarding other series, there was a higher frequency of *STAC3* and *CACNA1S* variants.

There were a few *RYR1* recurrent variants, which indicated a high genetic variability in the Brazilian sample, which is possibly the result of a population with a mixed ethnic background. Additionally, *RYR1* variants were distributed along the whole gene, not restricted to hot spots. Our data, together with data from other centers around the world, can contribute to identifying variants of unknown significance that are present in more than one family, which would be stronger candidates for future calcium release studies, allowing the promotion of these variants to pathogenic.

## Figures and Tables

**Table 1:** Patients with isolated heterozygous *RYR1* variants already described in malignant hyperthermia.

Patient	Variant	Criteria	Classification for AD-MH			Revel
		EMHG > VCEP > ACMG	EMHG	VCEP	This Work	
54	exon2:c.131G > A (p.Arg44His)	PS4_sup, PM1, PP3_mod	N/A	VUS	PP (PS4_mod, PM1, PP3_mod, PM5_sup) VECP	0.93
48	exon6:c:455C > A (p.Ala152Asp)	PS4_sup, PM1, PP3_mod	N/A	VUS	VUS (PS4_mod, PM1, PP3_mod)	0.9
52	exon6:c.487C > T (p.Arg163Cys)	PSb, PMb, PPa, PPb, PPc	P	P	-	0.96
34	**exon17:c.1840C > T (p.Arg614Cys)**	PSa, PMb, PPa, PPb, PPc	P	P	-	0.93
59	**exon17:c.1840C > T (p.Arg614Cys)**	PSa, PMb, PPa, PPb, PPc	P	P	-	0.93
4	exon29:c.4178A > G (p.Lys1393Arg)	BA1	N/A	B	-	0.56
11	exon39:c.6544A > T (p.Ile2182Phe)	PS4_sup, PM1	N/A	VUS	VUS (PS4_mod, PM1)	0.76
6	exon45:c.7292A > T (p.Asp2431Val)	PS4_sup, PM1_sup, PM5_sup, PP3_mod	N/A	VUS	PP (PS4_mod, PM1_sup, PM5_sup, PP3_mod)	0.95
31	exon 45:c.7300G > A (p.Gly2434Arg)	PSb, PMb, PPa, PPb, PPc	P	P	-	0.97
56	exon45:c.7304G > A (p.Arg2435His)	PSb, PMb, PPa, PPb, PPc	P	P	-	0.94
50	exon46:c.7358T > C (p.Ile2453Thr)	PS2_PM6_Mod, PS4_Sup, PM1, PP3_mod	N/A	PP	PP (PS2_mod, PS4_mod, PM1, PP3_mod)	0.89
14	exon46:c.7354C > T (p.Arg2452Trp)	PSb, PMb, PPa, PPc	P	PP	-	0.83
28	exon47:c.7523G > A (p.Arg2508His)	PSb, PMb, PPb, PPc	P	PP	-	0.9
9	**exon73:c.10747G > C (p.Glu3583Gln)**	BA1	N/A	B	B (BP4_sup, BA1)	0.32
20	**exon101:c.14545G > A (p.Val4849Ile)**	PSb, PMb, PPa, PPc	P	P	-	0.82
29	**exon101:c.14545G > A (p.Val4849Ile)**	PSb, PMb, PPa, PPc	P	P	-	0.82
40	exon104:c.14918C > T (p.Pro4973Leu)	PMb, PPb, PPc	PP	PP	-	0.9

Legend: EMHG: European Malignant Hyperthermia Group, VCEP: ClinGen MHS variant curation expert panel, MH: malignant hyperthermia, AD: autosomal dominant, N/A: not available, B: benign, VUS: variant of unknown significance, PP: probably pathogenic, P: pathogenic. Bold: recurrent variants.

**Table 2:** Patients with multiple *RYR1* variants.

Patient	Variant	Criteria	Classification for AD-MH			Revel
		EMHG/VCEP/ACMG	EMHG	VCEP	This Work	
1	exon101:c.14524G > A (p.Val4842Met) #	PM1_sup, PP3_mod	N/A	VUS	-	0.93
	intron:c.10348-6C > G (p.His3449ins33aafsX54) #	zero points; without applicable VCEP criteria	N/A	N/A	VUS for AD-MH (P for AR congenital myopathy)	0.67
12	exon33:c.4711A > G (p.Ile1571Val) #	BS1	N/A	PB	-	0.56
	exon67:c.10097G > A (p.Arg3366His) #	BS1	N/A	PB	-	0.68
	exon85:c.11798A > G (p.Tyr3933Cys) #	PP3_mod, BS1	N/A	PB	-	0.98
	**exon101:c.14545G > A (p.Val4849Ile)**	PSb, PMb, PPa, PPc	P	P	-	0.82
	intron:c.9555-9G > A	BP4_sup, BA1	N/A	N/A	PB	0.01
60	exon28:c.4055C > G (p.Ala1352Gly)	BP4_sup, BS2_mod, BA1	N/A	N/A	B	0.27
	exon71:c.10485G > C (p.Lys3495Asn)	zero points; without applicable VCEP criteria	N/A	N/A	VUS	0.58
5	**exon73:c.10747G > C (p.Glu3583Gln)**	BA1. BS2_SUP, BP4	N/A	B	-	0.32
	exon92:c.13459C > T (p.Leu4487Phe)	BP4	N/A	N/A	VUS	0.28
10	exon22:c.2767A > G (p.Met923Val)	zero points; without applicable VCEP criteria	N/A	N/A	VUS	0.69
	exon90:c.12532G > A (p.Gly4178Ser)	PS4_sup, PP3_mod	N/A	VUS	-	0.98
	exon92:c.13502C > T (p.Pro4501Leu)	BP4_sup, BA1	N/A	N/A	B	0.42
8	exon79:c.11314C > T (p.Arg3772Trp)—Homozygous	PS4_sup, PM5_sup, PP3_mod	N/A	VUS	-	0.94

Legend: EMHG: European Malignant Hyperthermia Group, VCEP: ClinGen MHS variant curation expert panel, MH: malignant hyperthermia, AD: autosomal dominant, N/A: not available, B: benign, PB: probably benign, VUS: variant of unknown significance, P: pathogenic. Bold: recurrent variants. #: haplotypes.

**Table 3:** Patients with novel isolated heterozygous *RYR1* variants.

Patient	Variant	Criteria	Classification for AD-MH			Revel
		EMHG > VCEP > ACMG	EMHG	VCEP	This Work	
17	exon6:c.425-19A > G	7BP4_mod, BS1_str	N/A	N/A	PB	0.02
33	exon6:c.452C > A (p.Pro151Gln)	PP3_sup, PM1_sup, PS4_sup	N/A	N/A	VUS	0.96
25	exon7:c.594A > G (p.Leu198Leu)	PM1, BP4_sup, BA1, BP7, BS2_str	N/A	N/A	B	0.1
7	exon20:c.2366G > A (p.(Arg789Gln)	zero points; without applicable VCEP criteria	N/A	N/A	VUS	0.81
46	Exon36:c.5999C > T (p.Ser2000Phe)	BP4	N/A	N/A	VUS	0.45
58	exon 44: c.7123G > C (p.Gly2375Arg)	PS1_mod, PM1_sup, PP3_mod, PS4_sup	N/A	N/A	PP	0.9
57	exon46:c.7354C > G (p.Arg2452Gly)	PM1_sup, PM5_mod, PS4_sup	N/A	N/A	VUS	0.79
45	**exon51:c.8197G > T (p.Gly2733Cys)**	PP3_mod, PS4_mod	N/A	N/A	VUS	0.87
37	**exon51:c.8197G > T (p.Gly2733Cys)**	PP3_mod, PS4_mod	N/A	N/A	VUS	0.87
47	exon84:c.11716A > G (p.T3906A)	BP4_sup	N/A	N/A	VUS	0.4
27	exon91:c.12828_12829insGAGGGCGCGGCGGGGCTC (p.G4284_T4285insAAGLEG)	PS4_sup	N/A	N/A	VUS	N/A
35	exon91:c.12629A > G (p.Lys4210Arg)	BP4_sup	N/A	N/A	VUS	0.34

Legend: EMHG: European Malignant Hyperthermia Group, MH: malignant hyperthermia, AD: autosomal dominant, N/A: not available, B: benign, PB: probably benign, VUS: variant of unknown significance, PP: probably pathogenic, Bold: recurrent variants.

**Table 4:** Variants in the *CACNA1S* and *STAC3*.

Patient	Variant	Gene	Criteria	Classification for AD-MH			Revel
			EMHG > VCEP > ACMG	EMHG	VCEP	This Work	
30	1. exon63: c.9472 + 30C > A	*RYR1*	BP4_sup	N/A	N/A	VUS	0.01
	2.exon39:c.4718C > T (p.Thr1573Met)	*CACNA1S*	zero points	N/A	N/A	VUS	0.42
43	**1.exon17:c.1840C > T** **(p.Arg614Cys)**	*RYR1*	PSa, PMb, PPa, PPb and PPc	P	P	-	0.93
	2.exon1:c.131G > A (p.Cys44Tyr)	*CACNA1S*	PM2_mod and PP3_sup	N/A	N/A	VUS	0.67
15	1.exon2:c.122T > C (p.Phe41Ser).	*RYR1*	PM1_mod and PP3_mod	N/A	N/A	VUS	0.93
	2 exon26:c.3256C > T (p.Arg1086Cys)	*CACNA1S*	PP3, PM2_sup, PM5, PS4_sup	N/A	N/A	VUS	0.96
2	c.851G > C (p.Trp284Ser)	*STAC3*	PP3, PM2, PS3_sup, PM3, and PP1_str	N/A	N/A	P for Myopathy with risk for MH AR	0.89
26	c.851G > C (p.Trp284Ser)	*STAC3*	PP3, PM2, PS3_sup, PM3, and PP1_str	N/A	N/A	P for Myopathy with risk for MH AR	0.89
3	c.851G > C (p.Trp284Ser)	*STAC3*	PP3, PM2, PS3_sup, PM3, and PP1_str	N/A	N/A	P for Myopathy with risk for MH AR	0.89

Legend: EMHG: European Malignant Hyperthermia Group, VCEP: ClinGen MHS variant curation expert panel, MH: malignant hyperthermia, AD: autosomal dominant, AR: autosomal recessive, N/A: not available, VUS: variant of unknown significance, P: pathogenic. Bold: recurrent variants.

**Table 5:** Clinical and laboratory findings in 61 patients with and without variants.

	Variant Absent (n = 20)	Variant Present (n = 41)	*p*
**Age**	29.5 ± 13.2	31.5 ± 17.9	0.61 *
**Sex (female/male)**	6/14	19/22	0.148 ^†^
**Ethnic background** **Caucasian** **Afro Brazilian**	13 (65%) 7 (35%)	30 (73.1%) 11 (26.8%)	0.431 ^†^
**Personal antecedents of MH during anesthesia**	7 (35%)	22 (53.6%)	0.17 ^†^
**CK (IU/L; n = 54)**	87 (64–157.5)	339 (162–563)	***p* < 0.0001 ^‡^**
**Muscle weakness**	2 (10%)	6 (14.6%)	0.253 ^†^
**Ptosis/strabismus**	4 (20%)	20 (48%)	**0.03 ^†^**
**Muscle hypertrophy**	9 (45%)	22 (53%)	0.403 ^†^
**Cores** **(n = 51)**	0 (0%)	8 (19.5%)	**0.013 ^†^**
**IVCT result (n = 50)** **MHShc** **MHSh** **MHSc**	4 (20%) 13 (65%) 3 (15%)	22 (73.3%) 4 (13.3%) 4 (13.3%)	**0.0002 ^†^** (MHShc vs. MHSh/MHSc, MHSh vs. MHShc/MHSc)
**Contracture 2 mmol caffeine**	0 (0–0.3)	1.6 (0.3–2.3)	***p* < 0.0001 ^‡^**
**Contracture 2% halothane**	0.3 (0.2–0.4)	2.2 (0.6–3.4)	***p* < 0.0001 ^‡^**

Legend: CK: creatine kinase, IVCT: in vitro contracture test, MHShc: contractures developed both to halothane and caffeine, MHSh: contractures developed only to halothane, MHSc: contractures developed only to caffeine. *: unpaired *t* –test, †: chi-square test, ‡: Mann–Whitney test.

## Data Availability

The original contributions presented in this study are included in the article and Supplementary Materials. Further inquiries can be directed to the corresponding author(s).

## Supplementary Materials

The following supporting information can be downloaded at: https://www.mdpi.com/article/10.3390/genes16101127/s1, Table S1. Clinical and laboratory findings.
