# Supplementary material for: Genetic Characteristics of Brazilian Patients with MH History
Source: Genes (Basel). 2025 Sep 25;16(10):1127. doi: 10.3390/genes16101127 (PMC12562892; doi:10.3390/genes16101127)
Supplement: Supplementary file 1 [file genes-16-01127-s001.zip › genes-3817995-supplementary.pdf]

Supplemental table S1. Clinical and laboratory data

Patient	Age	Sex	Ethny	MH	CK	Weakness	Ptosis/ Strabismus	Hypertrophy	Cores	IVCT	Subtype	Halothane 2%	Caffeine 2mMol
1	16	M	C	P	145	-	-	+	-	+	MHShc	1,24	0,28
2	12	M	AB	P	-	+	-	+	-	N/A	-	-	-
3	15	F	AB	P	-	-	+	+	-	N/A	-	-	-
4	8	M	AB	P	-	-	-	-	-	N/A	-	-	-
5	16	M	C	P	24815	-	+	+	-	N/A	-	-	-
6	24	M	C	P	320	-	-	+	+	+	MHShc	2,24	2,04
7	23	M	AB	P	208	-	+	+	-	+	MHSh	0,56	0
8	31	F	C	P	506	-	+	-	-	+	MHShc	2,12	2,4
9	20	F	C	P	37	-	+	+	-	+	MHSc	0	0,2
10	41	F	C	P	626	+	-	-	-	+	MHShc	4,56	4,36
11	23	F	AB	P	1061	-	-	-	-	+	MHShc	3,6	2,92
12	35	M	C	P	1519	+	+	-	+	+	MHShc	2,31	2,2
13	18	M	C	P	87	-	-	-	-	+	MHShc	0,52	0,2
14	18	M	C	F	621	-	+	+	+	+	MHShc	2,28	2,12
15	21	M	AB	P	384	-	+	-	-	+	MHShc	1,24	0,96
16	35	M	C	P	62	+	-	+	-	+	MHSc	0	0,32
17	30	M	C	P	136	-	-	-	-	+	MHSh	0,36	0
19	49	M	AB	P	328	-	-	+	-	+	MHShc	0,2	0,48
20	20	F	C	P	425	-	+	+	-	+	MHShc	2,72	0,8
21	26	M	AB	P	258	-	-	-	-	+	MHSh	0,48	0
22	16	M	C	P	181	-	-	-	-	+	MHSh	0,36	0
23	30	M	C	P	86	-	+	+	-	+	MHSh	1,16	0
24	35	M	AB	P	72	-	-	-	-	+	MHSh	0,36	0
25	45	M	C	P	140	-	-	+	-	+	MHShc	2,2	0,32
26	5	M	AB	P	-	-	+	+	-	N/A	-	-	-
27	18	F	C	P	90	+	-	-	-	+	MHShc	0,64	0,32
28	10	F	AB	P	1716	-	+	-	+	+	MHShc	4,4	3,72
29	54	M	AB	P	356	-	+	+	-	N/A	-	-	-
30	4	M	C	P	147	-	+	+	-	N/A	-	-	-
31	33	F	C	P	-	-	-	+	-	N/A	-	-	-

32	15	F	AB	F	37	-	+	+	-	+	MHSh	0,48	0
33	71	M	AB	F	373	-	+	-	+	+	MHShc	2,2	2
34	30	F	C	F	226	-	-	+	-	+	MHSh	0,4	0
35	17	F	C	F	105	-	-	-	-	+	MHSc	0	0,3
36	31	M	C	F	120	-	+	+	-	+	MHSh	0,6	0
37	52	M	C	F	547	-	+	+	-	+	MHShc	1,8	2,32
38	26	F	C	F	83	-	-	-	-	+	MHSh	0,28	0
39	55	M	AB	F	201	-	-	+	-	+	MHSh	0,2	0
40	51	F	C	F	96	-	-	-	-	+	MHShc	3,32	1,32
41	27	F	C	F	57	-	-	-	-	+	MHSh	0.2	0
42	45	M	C	F	66	-	-	-	-	+	MHSh	0.4	0
43	30	F	C	F	79	-	-	-	-	+	MHSh	1	0
44	43	F	C	F	48	-	-	-	-	+	MHSc	0	0,36
45	67	M	C	F	-	-	-	-	-	N/A	-	-	-
46	71	M	C	F	-	+	-	-	-	N/A	-	-	-
47	70	M	C	F	339	-	-	-	-	+	MHSc	0	0,4
48	43	M	C	F	579	-	+	+	+	+	MHShc	6,38	2,92
49	32	M	AB	F	168	-	-	-	-	+	MHShc	0,2	0,4
50	38	F	C	F	417,6	-	+	+	+	+	MHShc	3,44	1,96
51	26	M	C	F	74	+	-	+	-	+	MHSh	0,36	0
52	24	F	AB	F	195	-	+	+	-	+	MHShc	3,4	2,24
53	18	F	C	F	87	-	+	+	-	+	MHSh	0,24	0
54	44	F	C	F	303	-	-	-	-	+	MHShc	2,28	1
55	9	M	C	F	135	-	-	-	-	+	MHSc	0	0,4
56	42	F	C	F	376	-	-	+	+	+	MHShc	2,48	1,8
57	14	F	C	F	270	-	+	+	-	+	MHShc	3,84	1,84
58	33	F	C	F	179	-	+	-	-	+	MHShc	2,52	2,52
59	33	M	C	F	605	-	-	-	-	+	MHSc	0	1,04
60	14	M	C	F	190	+	-	+	-	N/A	-	-	-
61	40	M	C	F	52	-	-	-	-	+	MHShc	0.28	0.4
62	39	F	AB	F	110	-	-	+	-	+	MHSh	0,24	0

Legend: M-Male, F- Female, C- Caucasian, AB- Afro Brazilian, P- Personal, F- Family, MH- Malignant Hyperthermia, CK- Creatine Kinase, IVCT - in vitro contracture test, N/A: not available, MHShc- contractures developed both to halothane and caffeine, MHSh- contractures developed only to halothane, MHSc- contractures developed only to caffeine
